# The association between altered lipid profile and suicide attempt among Tunisian patients with schizophrenia

**DOI:** 10.1186/s12991-016-0123-1

**Published:** 2016-12-16

**Authors:** Rym Mensi, Amal Messaoud, Ahmed Mhallah, Islem Azizi, Walid Haj Salah, Wahiba Douki, Mohamed Fadhel Najjar, Lotfi Gaha

**Affiliations:** 1 Research Laboratory “Vulnerability to Psychotic Disorders LR05ES10”, Faculty of Medicine, University of Monastir, 5012 Monastir, Tunisia; 2 Department of Psychiatry, University Hospital in Monastir, Monastir, Tunisia; 3 Clinical Biochemistry and Toxicology Laboratory, University Hospital in Monastir, Monastir, Tunisia

**Keywords:** Lipids, Cholesterol, Schizophrenia, Suicide

## Abstract

**Background:**

There have been many studies on psychiatric disorders, but very little is known about the biology of suicide with schizophrenia. In the present study, we are looking for a possible connection between altered lipid profile and suicidal behavior in schizophrenic Tunisian patients.

**Methods:**

Assay of total cholesterol (TC), high-density lipoprotein cholesterol (HDL-c), low-density lipoprotein cholesterol (LDL-c), and triglycerides (TG) has been done for 126 schizophrenic patients with and without suicide attempts and 131 healthy controls recruited in the University Hospital of Monastir.

**Results:**

TC and LDL-c levels were significantly higher in schizophrenic patients compared to controls. TC was significantly lower in schizophrenic patients with suicide attempt compared to those without suicide attempt. Depending to the sonority of suicide attempt, TC was significantly lower in patients with recent suicide attempt compared to those with lifetime suicide attempt and without suicide attempt (*p* < 0.001), and no significant differences between TG, LDL-c, and HDL-c were noted.

**Conclusions:**

Results of this study showed that TC levels in schizophrenic patients after a recent suicide attempt are significantly lower than in patients without suicide attempt and with lifetime suicide attempts. TC can be one of biological markers defined suicidal risk for schizophrenic patients.

## Background

Suicide, a public health problem of high complexity with different etiological factors, is causing annually a premature loss of approximately one million lives worldwide [4] Suicide is the third most common cause of death in various countries in the 15–44 years age group, and the second most common cause of death in the 10–24 years age group (OMS [28]), and is still poorly understood. New approaches are therefore needed to complement the fundamental of social influences, cultural and individual propensity to suicide. Suicide is the chief cause of premature death among patients with schizophrenia [5]. The rate of suicide in schizophrenic patients is several times higher than in the general population. Identification of those at highest risk remains a problem for the clinician. Patients who have previously attempted suicide form a well-defined, high-risk group for suicide. It has been shown in other studies [16, 17, 39] that the prevalence of lifetime suicide attempts in females is almost twice than of males (7 vs. 4%, respectively), although the difference did not reach significant level.

In particular, improved prevention strategies are required in addition to extensive scientific study. Many researchers have focused on the search of biological markers that may be linked to suicidal behavior and can be used as an additional instrument for prevention and therapeutic action. It has been found that metabolic deregulation, especially altered lipid profile including low total cholesterol (TC) and low-density lipoproteins-cholesterol (LDL-c) levels, may underlie higher suicide risk in patients with schizophrenia [1, 3]. A number of studies have investigated a possible link between low serum cholesterol and psychiatric symptoms, especially suicidal behavior [36]. These findings might be explained by the hypothesis that reduced cholesterol level contributes to decreased serotonergic transmission due to altered affinity and function of serotonin receptors and transporters [8]. It has been further hypothesized that low peripheral and central cholesterol levels may reduce lipid viscosity of neuronal cell membranes lowering the availability of pre-synaptic serotonin transporters and post-synaptic serotonin receptors [21, 38]. However, it should also be noted that some studies have failed to prove an association between lipid profile and suicide in patients with schizophrenia [20, 22, 29, 34]. In sum, cholesterol has received attention as a potentially meaningful biomarker for suicide.

Most previous studies with clinical population had small samples, and focused on total cholesterol as the measure of interest. Given that each lipoprotein sub-fraction has a different role in the human body, total cholesterol might not be sufficient to determine the relationship between lipids and suicidality. Additionally, it is not clear whether a lower lipid profile is either a state or trait marker for suicide. Other blood markers should be compared in the same population to investigate potential biomarkers equally and to evaluate group differences between suicide attempters and non-attempters.

Therefore, in the present study, we investigated the possible connection between serum cholesterol, triglycerides, HDL-c and LDL-c levels, and suicide for schizophrenic patients with and without suicide attempts and healthy controls.

## Methods

### Patients

The protocol of this study was approved by the ethical committees of the University Hospital in Monastir. After providing consent, 126 subjects (91 men and 35 women) with the age range of 20–65 years were recruited during 11 months (April 2013–March 2014) at the Department of psychiatry, in the University Hospital of Monastir, which is located in the Mid-eastern part of Tunisia. We recruited patients randomly but only those that met the inclusion criteria for our study. Socio-demographic and clinical data were collected by an information sheet and from the medical record of the patient. We excluded all patients with metabolic disorders. The blood sample of the control group was taken from the blood bank of University Hospital in Monastir Tunisia; 131 Healthy controls (94 men and 37 women) did not have any mental or metabolic illness and they are in the same age range of patients (20–65 years). The suicide attempts were divided into two groups: lifetime suicide group (who had attempted suicide for more than 2 months) and recent suicide group (who had attempted suicide for less than 2 months). BMI was calculated as the weight (kilograms) divided by the square of the height (square meters).

### Assessment

Venipuncture was performed for all subjects between 8 and 9 a.m. after a 12 h overnight fast. Approximately 5 ml of blood was collected. Immediately after collecting blood samples, serum concentration of total cholesterol (TC), high-density lipoprotein cholesterol (HDL-c), and triglycerides (TG) were determined using enzyme methods on COBAS 6000™ automates analyzer, and low-density lipoprotein cholesterol (LDL-c) was calculated with the *Friedewald* equation. Reference intervals for the measured parameters were as follows: TC <5.0 mmol/L, LDL <3.0 mmol/L, HDL >1.0 mmol/L, and TG <1.7 mmol/L.

### Measurements

Table 1 shows comparison of socio-demographic details (age, gender, BMI, smoking status, and alcoholic status) between patients and controls.

**Table 1:** Demographic characteristics of different groups of study population

Demographic characteristics	±SD/freq. (%)		*p*
	Schizophrenia group (*n* = 126)	Controls group (*n* = 131)	
Gender			
Male	91 (72.2%)	94 (71.8%)	0.934
Female	35 (27.8%)	37 (28.2%)	
Sex ratio	2.6	2.54	
Age (year)	43.44 ± 10.60	40.81 ± 14.60	0.101
BMI (kg/m^2^)	26.25 ± 5.86	24.86 ± 3.82	*0.026*
Smoking status			
Smoking	73 (57.9%)	54 (41.2%)	0.713
No-smoking	51 (40.5%)	77 (58.8%)	
Weaned	2 (1.6%)	0 (0%)	
Alcoholic status			
Consumer	24 (19%)	14 (10.7%)	0.056
No-consumer	102 (81%)	117 (89.3%)	

The structured clinical interview for diagnostic and statistical manual of mental disorder-IV (DSM-IV) for schizophrenia was applied to assess the psychiatric status of individuals.

In the current study, we have two groups: schizophrenic patients and healthy controls. Schizophrenic patients are divided into schizophrenic patients without suicide attempts, schizophrenic patients with lifetime suicide attempts, and schizophrenic patients with recent suicide attempts.

### Psychometric scales

For all patients, we conducted a psychometric assessment through psychometric scales: PANSS (*Positive and Negative Syndrome Scale*) which enables the assessment of positive symptoms, negative, and general psychopathology, CGI (*Clinical Global Impression*) which allows assessment of the severity, the therapeutic index, and improvise patients under treatment, EGF (*Global Assessment of Function*), BPRS (*Brief Psychiatric Rating Scale*), and CALGAGY (*Depression Scale For Schizophrenia*).

### Data analysis methods

Data collected from medical, laboratory, and treatment records were noted and entered into a database: SPSS for the Windows 20.0 package program was used for analyses to compare the clinical and demographic characteristics variables between patients and healthy subjects.

All data are presented as the mean ± standard deviation (SD) or as n (%). Analysis of various, Chi square test, independent simple *t* test, was used for the comparisons whenever appropriate. A result of *p* < 0.05 was accepted as significant.

## Results and discussion

A total of 126 subjects with schizophrenic disorders were included in this study; 55 subjects had presented an episode of suicide attempt (15 had a recent suicide attempters and 40 had a lifetime suicide attempters) and 71 patients without any suicidal behavior. In this study, we have more males than females (Table 1).

BMI was significantly higher in schizophrenic patients compared to controls. The majorities of our patients smoke and do not consume alcohol.

Suicidal thoughts were reported by 37.4% of men and 60% of women with schizophrenia. The predominant type of schizophrenia was undifferentiated for patients without suicide attempt (47.88%) and paranoid for patients with suicide attempt (45.44%).The majority of the prescribed Antipsychotic Drugs are typical (83.1%). In this study, we found that differences in psychometric scores between patients with and without suicide attempt are significantly higher in schizophrenic patients with suicide attempts for PANSS (general, positive, and negative), CGI Severity and BPRS are significantly higher in schizophrenic patients with suicide attempts (Table 2).

**Table 2:** Clinical characteristics of schizophrenic patients with and without suicide attempts

	Patients without SA (*n* = 71)	Patients with SA (*n* = 55)	*p*
Psychometric evaluation			
PANSS general	35.25 ± 14.97	41.71 ± 15.86	*0.021*
PANSS positive	14.70 ± 6.83	19.80 ± 8.88	*<0.001*
PANSS negative	17.52 ± 8.93	20.76 ± 9.02	*0.046*
CGI severity	3.01 ± 1.61	3.31 ± 1.46	*<0.001*
CGI overall improvement	2.32 ± 1.20	2.22 ± 0.91	0.292
CGI therapeutic index	4.94 ± 3.49	5.20 ± 3.34	0.590
EGF	61.42 ± 18.21	53.27 ± 17.44	0.678
BPRS	53.49 ± 23.53	69.24 ± 25.24	*0.012*
Calgary	6.56 ± 10.11	8.31 ± 6.21	0.262
Type of schizophrenia			
Undifferentiated	35 (47.88%)	17 (30.90%)	0.113
Paranoid	23 (32.39%)	25 (45.44%)	
Disorganized	13 (18.30%)	13 (23.64%)	
Neuroleptics			
Typical	59 (83.1%)	47 (85.5%)	0.952
Typical and atypical	12 (16.9%)	8 (13.5%)	
Doses of chlorpromazine	990.07 ± 617.703	963.18 ± 652.663	0.814
Age at onset (year)	25.99 ± 8.39	25.26 ± 8.98	0.643

**Fig. 1:**
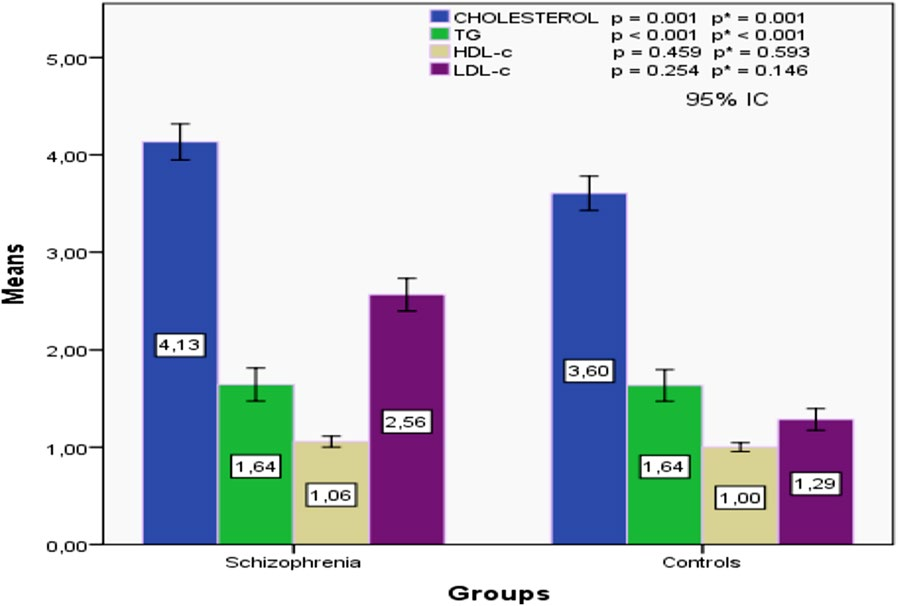
Plasma concentrations of lipid parameters in schizophrenic patients and controls. *ANOVA: p adjusted for BMI

Concentrations of total cholesterol and LDL-c were significantly higher in schizophrenic patients compared with controls. No relationship with HDL-c and triglycerides was found (Fig. 1).

Each lipid measure of the schizophrenic patients with recent suicide attempt, with lifetime suicide attempts, and without suicide attempt is shown in Table 3. The level of TC in the schizophrenic group with recent suicide attempts was significantly lower than schizophrenic groups with lifetime suicide attempts and without suicide attempts. There were no statistically significant differences in the LDL-c, HDL-c, and TG levels between these groups.

**Table 3:** Comparison of lipid profile in schizophrenic patients depending on the seniority of suicide attempt

Blood levels	Recent suicide attempters Group 1 (*n* = 15)	Lifetime suicide attempters Group 2 (*n* = 40)	Never-suicide attempters Group 3 (*n* = 71)	*p*	[1] vs. [3] *p*	[1] vs. [4] *p*	[3] vs. [4] *p*
TC (mmol/L)	3.19 ± 1.00	4.02 ± 0.96	4.39 ± 0.98	*0.000*	*0.007*	*0.000*	0.063
HDL-C (mmol/L)	1.66 ± 0.94	1.64 ± 1.04	1.64 ± 0.93	0.995	0.935	0.920	0.994
LDL-C (mmol/L)	1.04 ± 0.36	1.04 ± 0.27	1.04 ± 0.27	0.921	0.947	0.848	0.691
TG (mmol/L)	2.60 ± 0.50	2.62 ± 1.11	2.62 ± 1.12	0.847	0.934	0.746	0.597

The comparison reveals no differences in concentrations of TC, TG, HDL-c, and LDL-c between patients treated with typical, atypical, and association between these two classes of Neuroleptics drug (Table 4).

**Table 4:** Variations of lipid profile in schizophrenic patients according to the treatment

	Neuroleptics			*p*
	Typical [1]	Atypical [3]	Typical and atypical [4]	
TC (mmol/L)	4.16 ± 1.07	4.05 ± 0.25	4.05 ± 1.01	0.933
TG (mmol/L)	1.70 ± 1.41	1.41 ± 0.87	1.26 ± 0.64	0.210
HDL-C (mmol/L)	1.03 ± 0.28	1.21 ± 0.36	1.17 ± 0.48	0.145
LDL-C (mmol/L)	2.57 ± 0.98	2.25 ± 0.50	2.56 ± 0.72	0.797

Significant correlations between TC levels and EGF score as well as between the differences in TC levels and CALGARY score were found only for the subgroup of recent suicide attempters, indicating that TC levels may serve as a predictive factor for suicide attempts in patients with schizophrenia. There were also no associations between TC levels and score in PANSS (general, positive, and negative), CGI (severity, over all improvement, and therapeutic index), and BPRS (Table 5).

**Table 5:** Correlation of lipid profile in schizophrenic patients according to the psychometric evaluation for patients with recent suicide attempt

	TC (mmol/L)	TG (mmol/L)	HDL-c (mmol/L)	LDL-c (mmol/L)
PANSS general				
*r*	−0.050	−0.19	−1.17	0.04
*p*	0.575	0.830	0.191	0.967
PANSS +				
*r*	−0.11	−0.014	−0.093	0.076
*p*	0.901	0.875	0.301	0.398
PANSS −				
*r*	0.073	0.018	−0.020	−0.006
*p*	0.418	0.844	0.824	0.946
CGI severity				
*r*	−0.036	0.029	−0.142	0.039
*p*	0.689	0.749	0.114	0.664
CGI improving patients under treatment				
*r*	−1.06	0.051	−0.075	−0.006
*p*	0.237	0.569	0.404	0.945
CGI therapeutic index				
*r*	−0.057	−0.075	−0.090	−0.06
*p*	0.523	0.406	0.315	0.502
EGF				
*r*	−*0.186*	−0.058	−0.0514	−0.119
*p*	*0.037*	0.515	0.550	0.184
Calgary				
*r*	*0.222*	−0.046	0.132	0.150
*p*	*0.012*	0.609	0.139	0.093

**Fig. 2:**
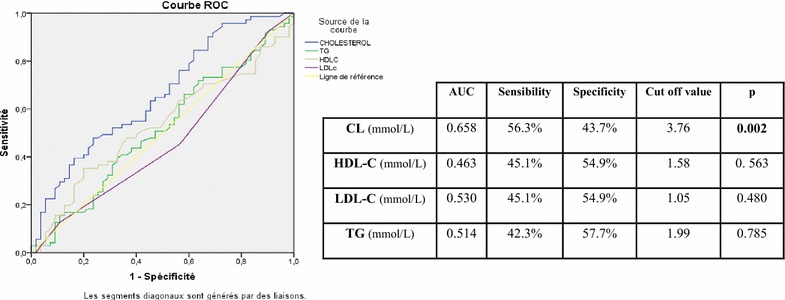
Roc curve assessing the different threshold values (TC, TG, HDL-c, and LDL-c) for patients with schizophrenia based on SA history

**Fig. 3:**
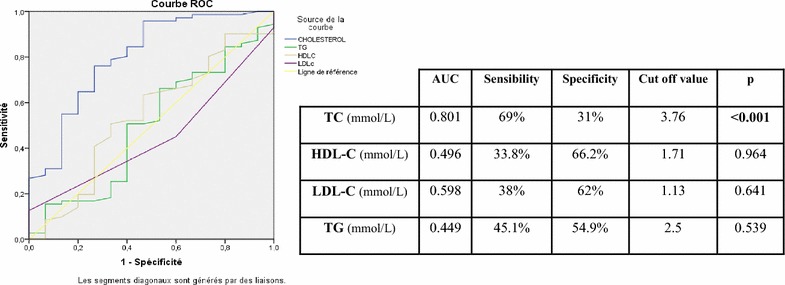
Roc curve assessing the different threshold values (TC, TG, HDL-c, and LDL-c) for patients with schizophrenia based on seniority of SA history

According to the threshold values represented by the Roc curve (Figs. 2 and 3), we divided our study of population to compare the effective of each interval. The results are summarized in Table 6.

**Table 6:** Total cholesterol, triglycerides, HDL-c, and LDL-c levels in schizophrenic patients with and without suicide attempters

	Schizophrenia		OR	Confidence interval 95%	*p*
	Without SA *N* = 71	With SA *N* = 55			
TC (mmol/L)					
≤3.51	12 (16.9%)	21 (38.2%)	*3.037*	1.330–6.932	*0.002*
>3.51	59 (83.1%)	34 (61.8%)			
TG (mmol/L)					
≤1.61	41 (57.7%)	34 (61.8%)	0.844	0.411–1.733	0.785
>1.61	30 (42.3%)	21 (38.2%)			
HDL-C (mmol/L)					
≤1.10	39 (54.9%)	36 (65.5%)	0.643	0.311–1.330	0. 563
>1.10	32 (45.1%)	19 (34.5%)			
LDL-C (mmol/L)					
≤2.5	39 (54.9%)	24 (43.6%)	1.574	0.775–3.198	0.480
>2.5	32 (45.1%)	31 (56.4%)			

In suicidal risk for patients with schizophrenia, the cutoff value of 3.76 mmol/L of TC was optimum, and at that point, sensitivity was 69% and specificity 31%. The area under the curve was statistically significantly lower in patients with a recent suicide attempt than for patients without suicide attempt (*p* < 0.001).

Schizophrenia is a serious mental disorder, and previous literature has reported that suicide was one of the main causes of premature death for sufferers from schizophrenia which was the major risk factor for complete suicide [32]. Cholesterol is a core component of the central nervous system (CNS), essential for the cell membrane stability, and the correct functioning of neurotransmission [33]. In our study, we found that TC was significantly higher among patients with schizophrenia compared to controls. Other studies have also shown that TC is higher among patients with schizophrenia compared to controls [25].

Considering the type of schizophrenia and according to our results and considering the type of schizophrenia, the majority of our patients with a suicide attempts are paranoid-type (41.81%), while the majority of patients without a suicide attempts are undifferentiated (46,47%), but the difference between the two types was not significant. The same results were found by Huang et al. [22].

The gender difference regarding risk of suicide attempt is well known. The well-known risk of attempted suicide is higher in females [31]. Accordingly, our study included more males than females with a suicide attempt. Higher frequency of alcohol consumption is found for patients (19%) compared to healthy controls (10.7%). In this study, alcohol consumption is less frequent compared to the results of North America or European studies which found high rates of use alcohol (rang 21–51%) [12]. These differences may be linked to availability of alcohol or cultural factors, in particular to the social pressure for alcohol abstinence in Tunisia as in other Islamic countries (Table 7).

**Table 7:** Total cholesterol, triglycerides, HDL-c, and LDL-c levels in schizophrenic patients with schizophrenia based on seniority of SA

	Schizophrenia		OR	Confidence interval 95%	*p*
	Lifetime SA	Recent SA			
TC (mmol/L)					
≤3.76	26 (36.6%)	12 (80%)	*6.923*	1.787–26.816	*<0.001*
>3.76	45 (63.4%)	3 (20%)			
TG (mmol/L)					
≤1.71	46 (64.8%)	9 (60%)	1.227	0.392–3.843	0.964
>1.71	25 (35.2%)	6 (40%)			
HDL-c (mmol/L)					
≤1.13	44 (62%)	11 (73.3%)	0.593	0.171–2.049	0.641
>1.13	27 (38%)	4 (26.7%)			
LDL-c (mmol/L)					
≤2.50	39 (54.9%)	6 (40%)	1.828	0.588–5.681	0.539
>2.50	32 (45.1%)	9 (60%)			

Therefore, the results obtained here corroborate most studies in which a relationship between suicidal behavior and low TC in schizophrenia patients was observed [26, 27]. Other studies did not find associations between serum cholesterol and death by suicide in patients with schizophrenia [35]. Some studies about attempted suicide mainly focused on patients suffering from major depression. Almeida-Montes et al. [2] found no significant difference in lipid profiles between patients who had attempted suicide and those who had not among patients with a diagnosis of a major depressive episode. No association between TC levels and suicidal act was shown in bipolar patients and those with major depressive disorder [8]. The same study showed that TG is significantly lower in those patients. The question has thus arisen as to why cholesterol levels have been found to be correlated with suicide attempts in some studies and not in others. Race differences in serum lipid profiles and lipoprotein lipase activity may be a culprit [15]; nutritional habits, life style, traditions, as well as seasons and climate may play a role in explaining these differences [10, 18, 19].

On the other hand, no association with Triglycerides, LDL cholesterol, and HDL cholesterol was observed in these patients for any lipidic parameter, which was also the case in the present study. However, Janusz et al. [23] have reported that total cholesterol, LDL cholesterol, total lipids, and triglycerides are significantly lower in patients with schizophrenia who had a suicidal attempt. In terms of gender differences, we must emphasize that females may be especially sensitive to dieting-induced changes in the central serotonin function [39].

Lower lipid levels in people with schizophrenia may also relate to the occurrence of metabolic syndrome. In relation to this, Vuksan-Cusa et al. [37] observed that the prevalence of metabolic syndrome in people with schizophrenia was lower in suicide attempters than in non-attempters. A post-mortem study reported down regulation of lipid metabolism mortem genes in the frontal cortex of suicide completers [24].

The aims of our study was to calculate the new cutoff value of total cholesterol according to the location of suicide in schizophrenic patients using Roc curve which was described by plotting the sensitivity on the y-axis against 1-specificity on the x-axis for each of several cutoffs. The results are interesting for total cholesterol in which 80% of patients with recent suicide attempt have a rate of TC ≤3.76 against 36.6% for patients without suicide attempt. Among patients who have not attempted suicide, 36.6% had rate of total cholesterol ≤3.76. Lipid profile of these patients should be controlled regularly to prevent suicide risk among them.

However, a unique measure of TC level may not reflect patient’s basic state. We cannot conclude if lower cholesterol is a state or a trait factor in suicide attempters. It is important to define an a priori biological threshold to distinguish subjects with low and high cholesterol. Additionally, since the study was conducted in a single university hospital, the finding may not be representative of all Tunisian patients with schizophrenia.

The absence of difference for triglyceride levels suggests that the association between cholesterol and suicidal risk is not influenced by nutritional status.

The effect of medication on cholesterol level is a potential confounder not taken into account in this study. Moreover, psychotropic drugs are usually associated with high cholesterol levels.

The relationship between low serum cholesterol levels and suicidal behavior is far from clear. There have been numerous hypotheses that have attempted to explain the mechanisms by which cholesterol levels may influence the risk of attempting suicide. The most widely held view is that there may be a link between serum cholesterol levels and the central nervous serotonergic system [7] via the reduction of brain serotonin activity [11]. Another hypothesis is that the phospholipids metabolism is disrupted, combining a deficit of incorporation of polyunsaturated fatty acids in the membrane with an increase their destruction [8]. Others have suggested that cholesterol may affect disease state and behaviors, as it plays a role in the production of the myelin sheath, in trans-membrane exchange, enzyme function, in the synthesis of steroid hormones, and neurotransmitter receptor expression [14].

However, it should be stressed that an association between low cholesterol and suicidal behavior in schizophrenia was not confirmed in a number of studies [22, 30]. Recently, Freemantle et al. [13] analyzed brain *oxysterol* levels, which are enzymatic oxidation products of cholesterol, in the prefrontal cortex of suicide victims. Their results show a significant increase in 24-hydroxysterol, reflecting a higher turnover of cholesterol. They suggest that this metabolic process may be responsible for a reduction in central and peripheral cholesterol in these subjects. These authors also found altered phospholipids levels connected with increased activity of *cholesteryl ester hydrolase*, which may impair inhibitory neurotransmission in the prefrontal cortex of subjects with violent suicides [13].

In subsequent studies, we will aim for the study of genes implicated in the transition to the suicidal act in patients with schizophrenia. We hypothesize that an array of alteration in genes responsible for lipid regulation is incremented in the suicide for schizophrenic patients.

As most of the studies yielding negative results in this respect were performed in Asian populations, it can be speculated that ethnic differences may play a role.

## Conclusion

The results show that total Cholesterol levels are low in suicide attempters after a recent suicide attempt, and remain lower than normal in a later time after the event.

Suicide prevention in patients with schizophrenia remains fundamental in the same way as the reduction of positive and negative symptoms, or improving the quality of life and reducing disability caused by the disease. We should automatically track a patient with schizophrenia suicidal risk factors or situations that promote their development.

One of the most important areas of research in suicide of schizophrenic patients should be based on the identification of biomarkers that may identify those patients prone to this risk of suicidal acts which is seemed important to focus on the specific involvement of fluidity membrane, and it is desirable to complete the analysis of lipid metabolism by assaying the various fractions of blood cholesterol to determine those included in this relationship.

## Limitations

The limitations of our study are the following: First, the sample size in the recent suicide attempt subgroup was small. It would be interesting to increase the sample size for this subgroup. The data for the current study were obtained from the region of Monastir. Future studies on this research topic may be conducted in larger and more diverse samples. Some variables, such as lipid total, were not measured in the present study, and this issue would be considered in the future investigation.

## Abbreviations

HDL-c: high-density lipoprotein cholesterol
LDL-c: low-density lipoprotein cholesterol
TC: total cholesterol
TG: triglycerides
SA: suicide attempt
